# Transactions of the Illinois State Dental Society

**Published:** 1887-10

**Authors:** 


TRANSACTIONS OF THE ILLINOIS STATE DENTAL SOCIETY.—This volume of proceedings contains many interesting and valuable articles some of which are illustrated, which were presented at the 22d annual meeting, held in Jacksonville, May 10th to 13th, 1887.

